# Posterior wedge osteotomy and debridement for Andersson lesion with severe kyphosis in ankylosing spondylitis

**DOI:** 10.1186/s13018-017-0556-5

**Published:** 2017-03-31

**Authors:** Yan Liang, Xiangyu Tang, Yongfei Zhao, Zheng Wang

**Affiliations:** Orthopedic Department, The General Hospital of Chinese People’s Liberation Army (301 hospital) Beijing, No.28.Fu Xing Rd, Hai Dian District, 100853 Beijing, China

**Keywords:** Osteotomy, Andersson lesion, Ankylosing spondylitis, Visual analog scale, Oswestry Disability Index

## Abstract

**Background:**

Andersson lesion is a well-known complication in ankylosing spondylitis. Recently, owing to the worry about the healing of fracture, some scholars advocated additional anterior surgery or other procedures were necessary, which increase the risk of the nerve injury. The purpose of this study is to introduce our experience and to explore the efficacy and feasibility of posterior wedge osteotomy and debridement through Andersson Lesion for surgical treatment of severe kyphosis in ankylosing spondylitis.

**Methods:**

From January 2012 to January 2014, a retrospective study of 14 Andersson lesion patients with severe kyphosis in ankylosing spondylitis treated with surgery was completed with an at least 2-year follow-up. The debridement procedure, before posterior wedge osteotomy in posterior approach, must scrape all sclerosis bone until healthy cancellous bone appears. Radiographic and clinical results and complications were assessed with an average follow-up of 24 months. The CT scan was obtained preoperatively and at the final follow-up to assess the displacement of the fracture preoperatively, the safety of screw insertion, the healing of the fracture at the final follow-up. The Bridwell interbody fusion grading system was used to assess the healing of the fracture.

**Results:**

Local kyphosis was substantially corrected from 51.7 ± 15.6 to 7.1 ± 19.5, with a mean correction of 44°. The global kyphosis (GK) changed from 60.6 ± 28.3 to 20.3 ± 10.3 (*P* = 0.000). The mean VAS back pain scores decreased from 6.7 ± 0.8 preoperatively to 0.75 ± 0.6 after a 2-year follow-up (*P* = 0.000). The ODI score improved from 60.56 ± 15.1% preoperatively to 23.46 ± 8.2% after a 2-year follow-up (*P* = 0.000). The CT scan showed solid fusion at the level of the AL, and no internal fixation loose. All patients achieved grade 1 fusion. No major complication occurred.

**Conclusions:**

The posterior wedge osteotomy and debridement through AL can be used to correct the severe kyphosis in ankylosing spondylitis, achieving favorable clinical outcomes, good fusion, and satisfactory deformity correction.

## Background

Andersson lesion (AL) is a destructive vertebral or discovertebral lesion that occurs in the late stage of the ankylosing spondylitis (AS), which is usually caused by a minor trauma. The exact prevalence of AL complicating AS in literature ranges from 1.5 to over 28% [1, 2]. It is a state of chronic mobile non-union with an essential posterior element fracture or unfused facet joints [3–5]. The lesion can result in back pain, neurologic deficits, and a progressive kyphotic deformity, which makes the treatment necessary.

The goals of surgical treatment are to achieve solid fusion, to decompress the nerve in patients with neurologic deficits, and to restore spinal alignment for patients with deformity [6]. However, the optimal procedure for this problem is still controversial [7]. Several surgical treatments for AL have been advocated, including posterior fusion, anterior fusion, and a combined approach [4, 8–11]. Owing to the worry about the healing of the fracture, some scholars advocated that the additional anterior surgery or other procedures were necessary, but such operation may increase the risk of the nerve injury. Due to the absence of definite evidence to prove that the single posterior osteotomy can obtain solid fusion and satisfactory deformity correction. The purpose of this study is to introduce our experience and to explore the efficacy and feasibility of the posterior wedge osteotomy and debridement through Andersson lesion for surgical treatment of severe kyphosis in ankylosing spondylitis.

## Methods

### Patients

From January 2012 to January 2014, 14 consecutive AL patients with severe kyphosis in AS treated with surgery in our hospital were retrospectively analyzed. They were followed-up at least 2 years. There were 13 males and 1 female, with an average age of 35.7 years old (range 22–44 years old). All patients complained of back pain and progression of the kyphosis deformity. All patients had participated in non-operative therapies, including bracing, resting, physiotherapy, and analgesics, without adequate relief of their symptoms before being considered for surgery.

### Study measures

Study measures were obtained by review of inpatient medical records and questionnaire. The primary measures of this study were blood loss, surgery time, visual analog score (VAS), and Oswestry Disability Index (ODI). Neurologic deficits were assessed according to the Frankel grading system.

### Radiologic assessment

Standing anteroposterior and lateral radiographs were performed preoperatively, postoperatively, and at the time of every follow-up. The measurement included local kyphosis (for the single level osteotomy, the local kyphosis was measured as the angle between the upper endplate of the vertebra one cephalad to the AL and the lower endplate of the vertebra one caudal to the AL. For the double level osteotomy, the local kyphosis was measured as the angle between the upper endplate of the vertebra one cephalad to the upper osteotomy and the lower endplate of the vertebra one caudal to the lower osteotomy), global kyphosis of the whole spine (GK), thoracic lumbar kyphosis (TLK), and lumbar lordosis (LL). Global kyphosis was measured between the maximally tilted upper and lower end vertebrae by using the standard Cobb’s method. Thoracic kyphosis was defined as the angle between the superior endplate of T5 and the inferior endplate of T12. Thoracic lumbar kyphosis was defined as the angle between the superior endplate of T10 and the inferior endplate of L2. Lumbar lordosis was measured between the superior endplate of T12 and S1. Besides, the pelvic parameters including sacrum slope (SS) and pelvic tilt (PT) were also measured. Magnetic resonance imaging (MRI) was used to identify cord compression. Patients with severe kyphosis could hardly enter the MRI machine, so MRI data were not collected in those cases. The CT scans of 14 patients were obtained preoperatively and at the final follow-up to assess the displacement of the fracture preoperatively, the safety of screw insertion, and the healing of the fracture at the final follow-up. The fusion criteria were based on Bridwell interbody fusion grading system (Table 1), and the assessments were performed by two independent assessors.

**Table 1:** Bridwell interbody fusion grading system

Grade	Description
I	Fused with remodeling and trabeculae present
II	Graft intact, not fully remodeled and incorporated, but no lucency present
III	Graft intact, potential lucency present at top and bottom of graft
IV	Fusion absent with collapse/resorption of graft

### Surgical procedures

Under general anesthesia, the patient was placed in a prone position on the operating table. The spine was exposed through a posterior midline approach with dissection laterally to the transverse process. Pedicle screws were inserted. Initially, laminectomy was performed at the site of pseudarthrosis. During the procedure of laminectomy, the dura was meticulously dissected. Then, the burr and the curette were used to debride the fibrous tissue and reactive sclerosis bone at the level of pseudarthrosis to expose normal cancellous bone surface. The debridement must be complete and careful. After that, wedge osteotomy was performed from the lateral vertebral wall to the midline according to the angle predetermined. Finally, the closure of osteotomy space was performed by gentle pressure on the pedicle screws above and below the osteotomy with precontoured rods to strengthen the stability of osteotomy site.

### Statistical analysis

Data were expressed as mean ± standard deviations for variables. Preoperative and postoperative differences were performed using paired *t* test, and the statistical significance was set at *P* < 0.05. All analyses were carried out by using the SPSS (Statistical Package for the Social Sciences) version 17.

## Results

### Surgical results

The average surgical time was 279.4 ± 32.9 min with a mean intraoperative blood loss of 1066.1 ± 466.7 ml. The level of Andersson lesion was T11/T12 in 5 cases (35.7%), T12/L1 in 5 cases (35.7%), and L1/L2 in 4 cases (28.6%). According to the angle predetermined preoperatively, 12 patients were operated with single level osteotomy, and 2 patients with double level osteotomy (Table 2).

**Table 2:** Patient demographics and operative data

Patient	Age (years)	Sex	Osteotomy level	Preoperative		2-year follow-up		Complication
				Frankel	LK (°)	Frankel	LK (°)	
1	27	M	2	Frankel E	35.9	Frankel E	−21.2	
2	22	M	1	Frankel E	41	Frankel E	2.8	
3	35	M	2	Frankel C	20.6	Frankel E	−44.6	
4	40	M	1	Frankel E	61.2	Frankel E	17.9	
5	44	M	1	Frankel D	30	Frankel E	6	
6	31	M	1	Frankel E	64.6	Frankel E	18.9	
7	39	F	1	Frankel E	66.4	Frankel E	23.8	Cerebrospinal fluid leakage
8	34	M	1	Frankel D	66.4	Frankel E	22.3	
9	38	M	1	Frankel E	67.4	Frankel E	23.2	Pneumonia
10	41	M	1	Frankel D	60.8	Frankel E	12.9	
11	35	M	1	Frankel E	45.1	Frankel E	1.8	
12	38	M	1	Frankel E	51	Frankel E	8	
13	33	M	1	Frankel E	45.4	Frankel E	2	
14	43	M	1	Frankel E	68.1	Frankel E	25.9	
Mean ± SD	35.7 ± 6.1				51.7 ± 15.6		7.1 ± 19.5	

### Clinical results

The mean VAS back pain scores decreased from 6.7 ± 0.8 preoperatively to 0.75 ± 0.6 after a 2-year follow-up (*P* = 0.000). The ODI score improved from 60.56 ± 15.1% preoperatively to 23.46 ± 8.2% after a 2-year follow-up (*P* = 0.000). All patients were satisfied with the surgical results. Four patients had incomplete neurological deficits (one patient with Frankel grade C and three with Frankel grade D) preoperatively had improved to Frankel grade E with normal strength and sensation at final follow-up (Table 3).

**Table 3:** Radiographic and clinical outcomes in 14 patients

Variables	Preoperative	2-year postoperative follow-up	*t*	*P*
LK	51.7 ± 15.6	7.1 ± 19.5	18.3	0.000 < 0.05
TLK	44.4 ± 12	6.6 ± 13.5	14.7	0.000 < 0.05
GK	60.6 ± 28.3	20.3 ± 10.3	7.5	0.000 < 0.05
LL	0.2 ± 25.6	−33 ± 15.7	10.4	0.000 < 0.05
SS	10.8 ± 14	25.7 ± 9.7	−7.5	0.000 < 0.05
PT	38.1 ± 14.8	20.2 ± 8	6.1	0.000 < 0.05
VAS	6.7 ± 0.8	0.75 ± 0.6	26.8	0.000 < 0.05
ODI	54.6 ± 12.8	15.2 ± 4.3	11.4	0.000 < 0.05

### Radiological results

**Fig. 1:**
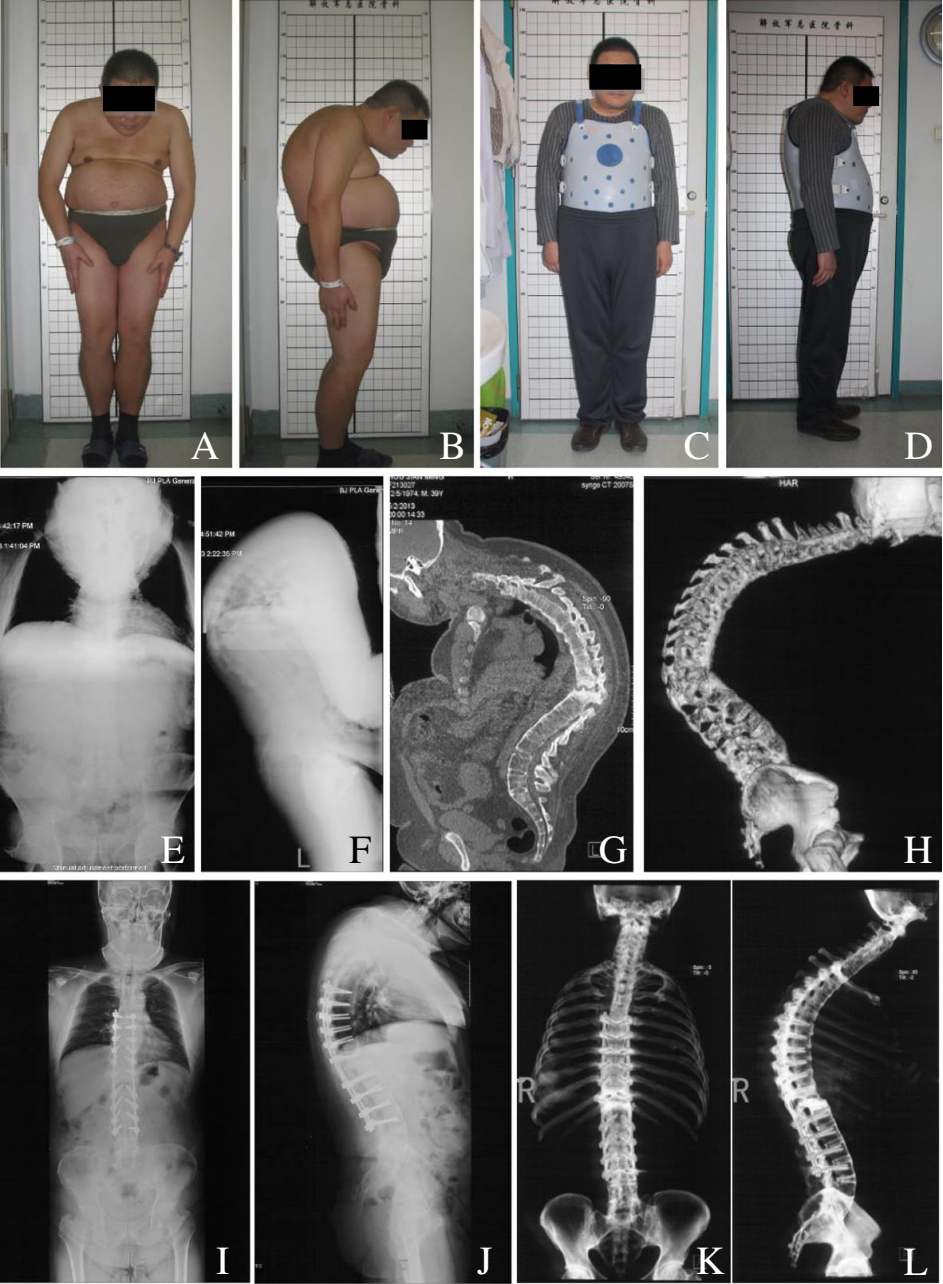
A 39-year-old male patient suffering from Andersson lesion-complicating ankylosing spondylitis. **a**–**d** Clinical appearance before surgery, and the final follow-up of 2 years. **e**, **f** Preoperative radiograph showed a remarkable kyphosis in thoracolumbar spine with a regional kyphosis of 72. **g**, **h** CT sagittal reconstruction image demonstrated fractures with irregular discovertebral osteolysis. **i**, **j** The X-ray at 2 years postoperatively showed the regional kyphosis was corrected to 17. **k**, **l** Three-dimensional reconstruction demonstrated that solid fusion of resection site was achieved

Local kyphosis was substantially corrected from 51.7 ± 15.6 to 7.1 ± 19.5, with a mean correction of 44°. The global kyphosis (GK) changed from 60.6 ± 28.3 to 20.3 ± 10.3 (*P* = 0.000), the thoracic lumbar kyphosis (TLK) changed from 44.4 ± 12 to 6.6 ± 13.5 (*P* = 0.000), and the lumbar lordosis (LL) changed from 0.2 ± 25.6 preoperatively to −33 ± 15.7 (*P* < 0.05) after a 2-year follow-up. The pelvic tilt (PT) decreased from 38.1 ± 14.8 preoperatively to 20.2 ± 8 after a 2-year follow-up (*P* = 0.000). The sacrum slope (SS) changed from 10.8 ± 14 preoperatively to 25.7 ± 9.7 after a 2-year follow-up (*P* = 0.000). All patients achieved grade 1 fusion after the final follow-up according to radiological evidence (Table 3) (Fig 1).

### Complications

There was one dura tear with cerebrospinal fluid leakage, which was repaired during operation, without other special treatment. One patient suffered from pneumonia and recovered after antibiotic treatment. There was no complication of neurologic injury, wound infection, or nonunion. There had been no breakage or failure of any screw or rod.

## Discussion

The AL was first described in 1937 [8]. Multiple possibilities for etiology of AL have been described, including infection, inflammation, trauma, and mechanical stress. According to the etiology, Cawley [12] classified these lesions into localized lesions and extensive lesions. For the inflammatory etiology, in the course of the disease, the extent of spinal inflammation and spinal fusion is not equally distributed over all vertebral or discovertebral segments. Local areas with increased spinal inflammation and decreased spinal fusion permit an excessive degree of mobility, resulting in a local nonunion of the ankylosed spine [13]. For the trauma etiology, in the complete ankylosed spine, a fracture will be the only moving segment between the long lever arms. Persistent motion at the fracture site may hinder fracture from healing and union, resulting in a sclerotic unfused spinal segment. The thoracolumbar and lumbar spine has disc spaces which is the most susceptible to shearing or distraction under the effect of gravity in the kyphotic spine, the most common site of AL.

Conservative treatment with brace, rest, and physiotherapy can be effective [14]. But it may result in progressive thoracolumbar kyphotic deformity, sagittal imbalance, intractable pain, and neurological deficit [3, 8, 9, 15]. Surgical instrumentation and fusion is considered to be the principle management in symptomatic AL that fails to resolve from a conservative treatment [8, 9, 16]. The goal of surgery is to decompress the canal and to restore spinal stability to facilitate the healing and fusion of the spinal lesion. However, the optimal surgical procedure is still in debate.

In the early stage, most surgeons recommended anterior fusion for AL with kyphotic deformity in patients with AS. They believe that anterior fusion allows them direct access to the anterior lesion and that it is biomechanically superior to posterior fusion in a kyphotic spine. Fang [4] suggested an anterior approach for SP in AS, and the approach achieved good results. But it is difficult to perform spinal deformity correction according to the anterior approach. In order to solve such problem, most surgeon advocated a combined anterior and posterior approach for the treatment of pseudarthrosis with kyphotic deformity in advanced AS patients [9, 17]. Chen [9] adopted anterior debridement and fusion with autograft for the pseudarthrosis in the first stage, followed by posterior fusion and instrumentation in the second stage, and successful fusion and good clinical result were achieved. Qian [10] recommended posterior osteotomy through pseudarthrosis was combined with anterior debridement, and bone graft fusion and great success was achieved. However, this two-step procedure increases the overall operation time and more blood loss, which lead to higher potential for surgical complications.

Recently, the single posterior approach to deal with AL is becoming more and more popular. Chang [8] proved that anterior fusion was not necessary because of the excellent reunion ability of AS. But the evidence of the healing of fracture was insufficient, and the deformity correction was not explicit. Zhang [16] adopted transpedicular subtraction and disc resection osteotomy technique to treat AL and achieved satisfactory outcomes. Wang [18] used a single posterior approach to debride the pseudarthrosis and perform bone graft to treat such patients, and achieved solid fusion. But the deformity correction was not mentioned in this article.

In this study, 14 AL patients with severe kyphosis in AS were treated with the posterior debridement and wedge osteotomy, and great success was achieved. For the treatment of AL in AS, two points are necessary: fusion and correction. In order to achieve solid fusion, the debridement and instrument are important. The surrounding of pseudarthrosis is filled with fibrous tissue and reactive sclerosis [8, 9, 16, 18]. If these tissues are not removed, the pseudarthrosis cannot be fused. So we used the burr and curette to resect the reactive sclerosis to expose normal cancellous bone surface. It is a necessary step to achieve solid fusion. For the patients with kyphotic deformity, kyphotic correction is another important procedure. Due to the existence of pseudarthrosis, the chosen level of the osteotomy procedure is limited by the Andersson lesion plane. According to the osteotomy angle designed preoperative, we used the closing wedge osteotomy (CWO) or closing-opening wedge osteotomy (COWO) to correct the kyphotic deformity. After the debridement and osteotomy, the middle and posterior columns of vertebrate with or without anterior column contact with each other, which is fused well because of the excellent reunion ability of AS. The outcome of CT scan showed that good fusion was obtained at the time of follow-up. Compared to other posterior method to treat AL, the cage and iliac crest bone graft are not needed, which lead to less operative risk, including less blood loss and less operating time.

We used the posterior wedge osteotomy and debridement through AL to correct severe kyphosis in AS, achieving satisfactory deformity correction and good fusion at the same time. With an exception to explore the fusion situation by the posterior surgery, the purpose of this study is also to show how much correction can be obtained with wedge osteotomy at the level of the AL. In our study, the local kyphosis was substantially corrected from 51.7 ± 15.6 to 7.1 ± 19.5, with a mean correction of 44°. The GK changed from 60.6 ± 28.3 to 20.3 ± 10.3 (*P* = 0.000), the TLK changed from 44.4 ± 12 to 6.6 ± 13.5 (*P* = 0.000), and the LL changed from 0.2 ± 25.6 to -33 ± 15.7 (*P* = 0.000). The PT decreased from 38.1 ± 14.8 to 20.2 ± 8 (*P* = 0.000). The SS changed from 10.8 ± 14 to 25.7 ± 9.7 (*P* = 0.000). Such data indicated that the sagittal balance and the quality of life were improved by the posterior wedge osteotomy at the level of the AL. No notable loss of any correction occurred at the site of osteotomy. So, the single posterior surgery can achieve satisfactory deformity correction in the treatment of the AL with severe kyphosis in AS.

In summary, the posterior wedge osteotomy and debridement through AL can successfully treat severe kyphosis in AS. As the number of the cases are small, the larger sample research is needed for further study.

## Conclusions

For the patients of AL with severe kyphosis in AS, the posterior wedge osteotomy and debridement can achieve favorable clinical outcomes, good fusion, and satisfactory deformity correction. Sacrum slope (SS) and pelvic tilt (PT).

## Abbreviations

AL: Andersson lesion
AS: Ankylosing spondylitis
GK: Global kyphosis
LL: Lumbar lordosis
TLK: Thoracic lumbar kyphosis
VAS: Visual analog score
